# Disclosing the Interaction between Carbon Monoxide and Alkylated Ti^3+^ Species: a Direct Insight into Ziegler–Natta Catalysis

**DOI:** 10.1021/acs.jpclett.0c01665

**Published:** 2020-06-25

**Authors:** Alessandro Piovano, Jelena Zarupski, Elena Groppo

**Affiliations:** Department of Chemistry, INSTM and NIS Centre, University of Torino, Via Giuria 7, 10125 Torino, Italy; DPI, P.O. Box 902, 5600 AX Eindhoven, The Netherlands

## Abstract

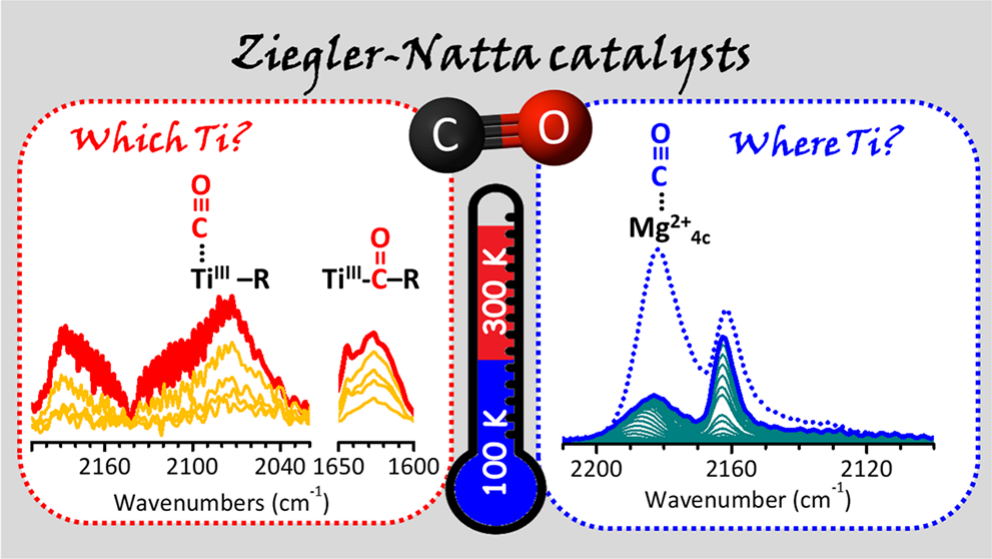

In the field of Ziegler–Natta catalysis for olefin polymerization, carbon monoxide (CO) is used in the industrial practice to quench the reaction when it proceeds too fast, approaching critical levels for the plant safety. The quenching effect is explained as due to the reversible coordination of CO to the titanium active sites, but no direct evidence has been ever reported. In this work, we designed a series of experiments to monitor CO adsorption at variable temperatures on a model Ziegler–Natta catalyst by means of FT-IR spectroscopy. For the first time, we have been able to spectroscopically detect CO coordinated to alkylated Ti^3+^ sites and the Ti–acyl species formed upon the subsequent insertion of CO into the Ti^3+^–alkyl bond, both in the absence and in the presence of the olefin monomer. In perspective, this has important implications for the characterization of the active sites in industrial Ziegler–Natta catalysts, even under working conditions.

Synthetic polymers are nowadays one of the main features of anthropogenic activities, emblematic of the continuously evolving society and of the growing human impact on the planet. More than 60% of these materials is constituted by polyolefins, whose widespread development has been drastically increasing since 1950s, i.e., since the almost simultaneous inventions of Ziegler–Natta and Phillips catalysts, which are still the most used in the field.^1,2^ Ziegler–Natta catalysts are heterogeneous multicomponent and hierarchical systems, whose composition has been optimized over the years mainly through a trial-and-error approach, passing across many different generations.^3,4^ Nowadays, they are constituted by four main components interconnected with each other.^5^ The precursors of the catalytic sites are TiCl_*x*_ species chemisorbed on a nanostructured high-surface-area MgCl_2_, which contributes to the stereoselectivity of the catalyst.^6−10^ Organic electron-rich compounds (called electron donors) are usually added to the catalyst to improve the productivity and stereospecificity.^4,10,11^ The TiCl_*x*_ precursors need to be activated by an Al–alkyl cocatalyst before being effective in olefin polymerization. According to the well-established Cossee–Arlman mechanism, the cocatalyst has the double role of reducing and alkylating the titanium centers, replacing a chlorine ligand with an alkyl group and creating a coordinative vacancy, which is essential for the subsequent insertion of the monomer.^12^

Some fundamental questions about the structure and working of Ziegler–Natta catalysts are still open, setting the bar for the rational design of new and more efficient processes. Spectroscopic techniques have the potential to make up for this lack, unveiling the different species on the catalyst and monitoring their evolution during the catalyst synthesis and under reaction conditions. This is valid in general for heterogeneous catalysts,^13^ and it has been recently demonstrated to be a valuable approach also for characterizing Ziegler–Natta catalysts through a step-by-step approach, so as to isolate the contribution of each component, thus overcoming the intrinsic complexity of the catalyst.^14^ In this respect, FT-IR spectroscopy of adsorbed CO has been proved to be a powerful technique to probe the MgCl_2_ surface as well as the distribution of the titanium species in Ziegler–Natta precatalysts,^15−17^ and more recently it has been used to assess the overall condition of some industrial Ziegler–Natta catalysts and predict their effectiveness in catalyzing olefin polymerization.^18^

Potentially, the use of a CO probe might be further exploited to get direct information on the titanium active sites and their structure and reactivity, as widely done for many other heterogeneous catalysts,^19,20^ including Cr-based catalysts for olefin polymerization.^21,22^ As a matter of fact, CO is used in the polyolefin industry to temporarily quench the polymerization reaction when it is running too fast to reach critical conditions for the plant.^23,24^ The ability of CO to poison Ti-based catalysts in a reversible way was used since the beginning as one of the principal evidence of coordinative unsaturation of the active titanium centers.^25^ The CO interaction interrupts the polymerization reaction until CO is slowly removed by the cocatalyst or the monomer itself, so that the initial catalytic activity is restored.^26^ Following this principle, the CO inhibition effect was used traditionally as one of the methods to determine the amount of active sites in Ziegler–Natta catalysts, by evaluating the CO/Ti ratio that must be injected into the reactor to completely stop the gas-phase polymerization reaction.^27−30^ Moreover, the use of CO labeled with ^14^C and ^13^C demonstrated that the coordinated CO molecules further react with the active centers, inserting into the Ti–alkyl bonds and generating carbonyl groups, detected into the CO-quenched polymer chains.^31,32^ Tritto et al. investigated this reaction, contacting a Ziegler–Natta catalyst with CO in the absence of any monomer and hydrolyzing the catalyst with HCl: in the extracted organic compounds they revealed the presence of carbonyls and their derivatives, thus confirming the rearrangement of CO with the alkyl groups of the activated titanium centers even in the absence of the monomer.^33^

However, no information has ever been achieved about the direct bond between Ti and CO, because of a combination of experimental difficulties, as the low amount of active sites, their strong sensitivity to poisons, and, quite important, their weak interaction with CO. Only recently Pletcher and co-workers have exploited CO as a direct characterization tool for an industrial Ziegler–Natta catalyst, monitoring CO adsorption at 100 K with FT-IR spectroscopy to assess the catalyst deterioration under different environmental conditions.^18^ From the deconvolution of the complex ν(C≡O) signal they tentatively assigned a band at 2091 cm^–1^ to CO in interaction with Ti^3+^ sites,^18^ but a deeper investigation of that interaction was out of topic for that study, and a more accurate description is still missing. Herein, we address this point by designing and performing a series of FT-IR experiments aimed at pursuing the few titanium sites active in ethylene polymerization in a model Ziegler–Natta catalyst.

The catalyst was synthesized as discussed in ref (14), where each step of the synthesis was characterized by multiple spectroscopic techniques. Briefly, a MgCl_2_–6MeOH precursor was prepared by azeotropic distillation^34^ and successively dealcoholated under a dynamic vacuum at 200 °C to get a high-surface-area MgCl_2_ (100 m^2^/g).^17^ Afterward, TiCl_4_ was dosed from the vapor phase and heated at 90 °C for 1 h to promote its chemisorption on MgCl_2_ surface. The excess of TiCl_4_ was outgassed at the same temperature under dynamic vacuum. The so formed MgCl_2_/TICl_4_ Ziegler–Natta precatalyst contains ca. 1 wt % of Ti.^17^ Finally, the MgCl_2_/TiCl_4_ precatalyst was activated by triethylaluminum (TEAl) at room temperature from the vapor phase, at an Al:Ti ratio of 1:1. The final Ziegler–Natta catalyst will be hereafter referred to as MgCl_2_/TiCl_4_/TEAl. Although this synthesis procedure differs from the standard industrial routes, it has the advantage to be performed altogether inside the same measurement cell, allowing us to monitor the changes occurring in the sample by FT-IR spectroscopy of adsorbed CO at each step of the synthesis.

In a first series of experiments, the surface changes were probed by CO adsorption at 100 K. Figure 1 shows three sequences of spectra, in the ν(C≡O) region, collected at 100 K as a function of the CO coverage (θ), for CO adsorbed on MgCl_2_ (part a), MgCl_2_/TiCl_4_ (part b), and MgCl_2_/TiCl_4_/TEAl (part c). Although spectra analogous to those shown in Figure 1a,b were already discussed in our previous work,^17^ they are here reported for the sake of comparison and briefly discussed in the following. When CO is dosed at 100 K on MgCl_2_ (Figure 1a), two bands are observed at 2182 and 2163 cm^–1^ (at the maximum coverage, θ_max_), the former more intense and more resistant to degassing than the latter. They have been assigned to CO adsorbed on strongly acidic and weakly acidic Mg^2+^ sites, respectively, which are specific of the different exposed surfaces.^17^ For MgCl_2_ obtained from the conversion of an alcoholate precursor, the relative extent of the exposed surfaces depends on the alcohol.^16,35,36^ When the alcohol is methanol, the surfaces exposing strongly acidic Mg^2+^ sites (i.e., (110), (015), and (012)), are the most favored. In the spectrum of CO adsorbed on the MgCl_2_/TiCl_4_ precatalyst (Figure 1b) the band at 2182 cm^–1^ is drastically less intense than before, while the band at 2163 cm^–1^ is almost unchanged. This was taken as an indication that the strongly acidic Mg^2+^ sites are no more available for CO adsorption because they are selectively occupied by chemisorbed TiCl_*x*_ species,^17^ in agreement with recent theoretical calculations.^37−41^

**Figure 1:**
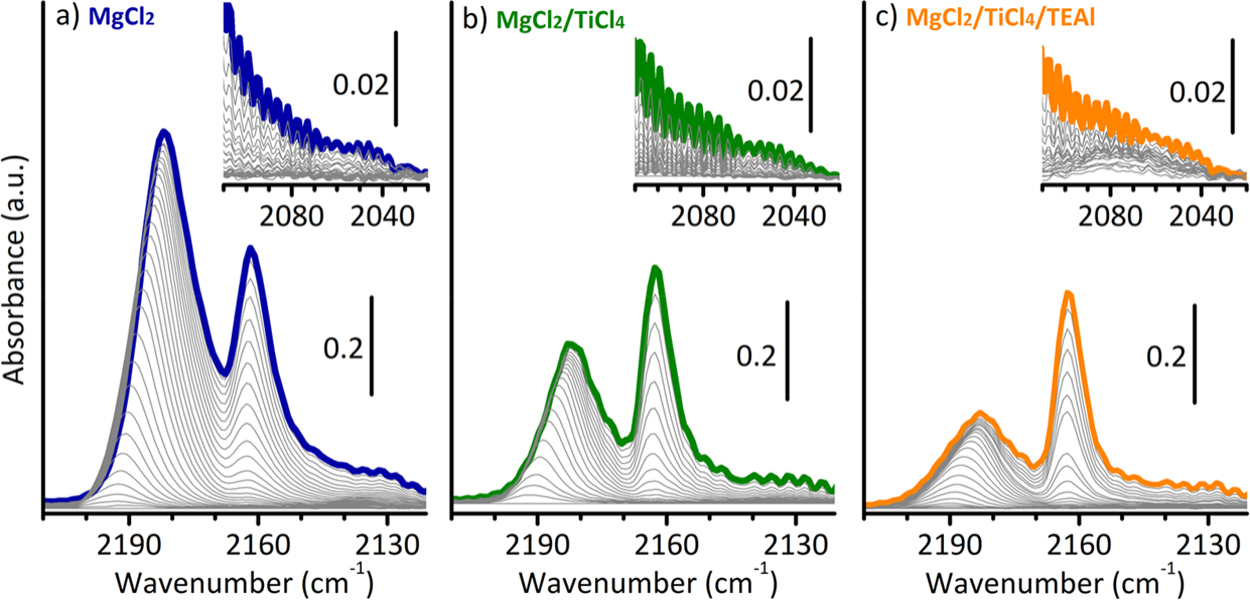
FT-IR spectra in the ν(C≡O) region of CO adsorbed at 100 K on MgCl_2_ (a), MgCl_2_/TiCl_4_ (b), and MgCl_2_/TiCl_4_/TEAl (c), as a function of CO coverage (θ_max_ in bold colors). The insets show a magnification of the 2110–2020 cm^–1^ region. The spectra are shown after subtraction of spectrum of the catalyst prior CO dosage.

After activation of MgCl_2_/TiCl_4_ precatalyst by TEAl (Figure 1c), the FT-IR spectrum of CO adsorbed at 100 K does not show significant changes in the distribution of the accessible surface sites. The band at 2163 cm^–1^ is almost unaffected, while that at 2182 cm^–1^ slightly decreases in intensity with respect to the precedent step. As a matter of fact, although TEAl does not selectively occupy any specific site at MgCl_2_ surface, after reaction with the chemisorbed TiCl_*x*_ species the so formed diethylaluminum chloride (DEAC) byproduct likely adsorbs on MgCl_2_ surface in close proximity of the Ti center.^42^ No additional bands due to the interaction of CO with aluminum cations are detected, which would have been expected over 2200 cm^–1^.^43^ However, by carefully looking at the sequence of the spectra as a function of CO coverage, a very weak band can be noticed at around 2076 cm^–1^, not present in the previous spectra and very resistant to the lowering of CO pressure (inset in Figure 1c). A band at a similar position was attributed by Pletcher et al. to CO interacting with Ti^3+^ sites,^18^ although the same authors warned about the possible misunderstanding with the signal of CO in interaction with the Mg^2+^ sites through the O atom.^44^

In order to better clarify the situation, CO adsorption on the MgCl_2_/TiCl_4_/TEAl catalyst was repeated at room temperature. Indeed, at room temperature the interaction of CO with the bare MgCl_2_ surface is almost negligible, while the CO inhibiting effect on the titanium centers is effective even at 60–100 °C, which is the typical temperature of industrial olefin polymerization. Because of the very low intensity of the band under scrutiny, an instrumental setup with a very short optical path was adopted, to minimize the roto-vibrational contribution of CO in the gas phase.

Figure 2a reports the spectrum of CO adsorbed at room temperature on the MgCl_2_/TiCl_4_/TEAl catalyst and its evolution upon lowering θ. At θ_max_, the spectrum (spectrum 1) is characterized by two absorption bands at 2185 and 2076 cm^–1^, springing out from the roto-vibrational profile of CO gas. The former corresponds to a small fraction of CO molecules adsorbed on the strongly acidic Mg^2+^ even at room temperature.^17^ The latter recalls what is already reported in the inset of Figure 1c, but much more resolved. The observation of this band even when CO is adsorbed at room temperature on MgCl_2_/TiCl_4_/TEAl allows discarding the hypothesis of CO O-bonded to Mg^2+^ cations, which can be observed only when CO is adsorbed at 100 K. Hence, we definitely ascribe it to CO in interaction with Ti^3+^ sites. It is worth noticing that the position of the band is the result of a balance between the polarizing effect of Ti^3+^ ions, which have a Lewis acid nature, and the π-back-donation due to the unpaired electron in the external d orbital of the reduced titanium cation. This band decreases rapidly upon decreasing the CO pressure, indicating that the interaction of CO with the Ti^3+^ sites is quite weak at room temperature and fully reversible.

**Figure 2:**
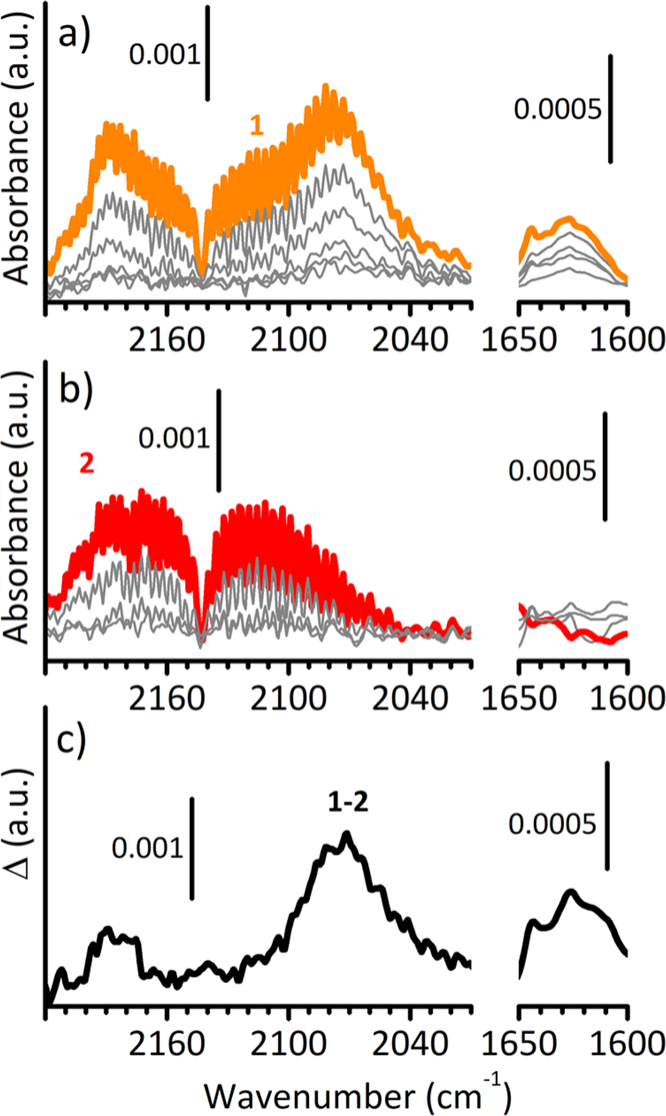
(a) FT-IR spectra of CO adsorbed at room temperature on MgCl_2_/TiCl_4_/TEAl as a function of CO coverage (θ_max_ in orange, spectrum 1). The spectra are shown after subtraction of that of the catalyst prior CO dosage. (b) Same as in part a, but after exposing MgCl_2_/TiCl_4_/TEAl to O_2_ (θ_max_ in red, spectrum 2). (c) Difference spectrum obtained by subtracting spectrum 2 to spectrum 1.

Finally, a very weak feature is also detected at ca. 1625 cm^–1^. This band is assigned to ν(C=O) of a Ti—acyl species formed upon insertion of CO into the Ti—alkyl bond, similarly to what happens in carbonylation reactions.^45^ This observation is in agreement with the previously cited literature reporting the presence of carbonyl groups in the CO-quenched polymer chains.^31,32^ As a matter of fact, CO insertion into a metal–carbon σ bond is well documented for most of 3d, 4d, and 5d elements, and it has been demonstrated to proceed by alkyl migration,^46^ in the same way as olefin insertion in Cossee–Arlman mechanism.^12^ Also this band is reversible upon decreasing the CO pressure, although at a slower rate with respect to CO coordinated to Ti^3+^ sites. This behavior indicates that the removal of CO from the Ti—C bond requires the restoration of the coordination vacancy at the Ti site and is a direct evidence that the reverse reaction, i.e., CO insertion into the Ti—C bond, proceeds through a coordination stage.

In order to further verify the nature of the bands at 2076 and 1625 cm^–1^, CO adsorption on the MgCl_2_/TiCl_4_/TEAl catalyst was repeated after exposing the sample to oxygen, with the intention to oxidize the accessible Ti^3+^ sites making them no more visible by CO. As a matter of fact, it has been reported that the accessible Ti^3+^ in Ziegler–Natta catalysts reacts with O_2_ via an electron-transfer mechanism to give an O_2_^–^ superoxo radical in interaction with Ti^4+^.^47^ The spectra are reported in Figure 2b as a function of θ, while Figure 2c shows the difference spectrum obtained by subtracting spectrum 2 to spectrum 1, emphasizing the effect of O_2_. As expected, at θ_max_ (spectrum 2) the absorption bands at 2076 and 1625 cm^–1^ are not present anymore. Interestingly, also the band at 2185 cm^–1^ is no more visible. This suggests that the few strongly acidic Mg^2+^ cations previously probed by CO are no more accessible after exposure of the catalyst to O_2_, likely because O_2_^–^ superoxo species or their derivatives are adsorbed also on Mg^2+^ sites, as demonstrated to occur on defective MgO.^48−50^ Evidence for the formation of O_2_^–^ superoxo species on the MgCl_2_/TiCl_4_/TEAl catalyst are present in the (not shown) FT-IR spectrum of the oxidized catalyst, displaying a broad absorption in the 1150–1000 cm^–1^ region, which is the spectral region characteristic for the ν(O–O) vibration of adsorbed superoxo species,^51^ but not well-defined because of both the heterogeneity of the reactive sites and the possible evolution paths for the newly formed adsorbates.

A final experiment was performed to probe with CO the Ti^3+^ species during ethylene polymerization. To this aim, a mixture of CO and ethylene (equilibrium pressure = 100 mbar, 1:1 molar ratio) was dosed on MgCl_2_/TiCl_4_/TEAl, mimicking the condition experienced by the catalyst in the industrial plant when CO is used to avoid the reactor fouling.^23,24^ The results are shown in Figure 3, which reports the FT-IR spectra collected when the catalyst is exposed to the C_2_H_4_/CO mixture (part a), during the subsequent degassing (part b), and finally after exposing it back to pure ethylene (part c). The spectra are reported in three spectral regions, corresponding to ν(CH_2_), ν(C≡O), and ν(C=O). As soon as the C_2_H_4_/CO mixture is introduced into the reaction cell (Figure 3a) ethylene polymerization immediately starts, as demonstrated by the fast appearance of two bands at 2920 and 2852 cm^–1^, assigned to the asymmetric and symmetric ν(CH_2_) modes of PE.^52^ However, the reaction rapidly stops after 40 s, along with the coordination of CO to the Ti sites (band at 2072 cm^–1^) and the insertion into the Ti—alkyl bond (band at 1620 cm^–1^). Interestingly, both the ν(C=O) of Ti—acyl and the ν(C≡O) of CO adsorbed on Ti^3+^ sites are slightly downward shifted with respect to what is observed upon adsorption of CO on MgCl_2_/TiCl_4_/TEAl in the absence of ethylene (Figure 2a). The shift of the former band may be caused by different spatial dispositions of the Ti—acyl species as hypothesized for the carbonylation of organotitanium complexes,^45^ while the shift of the latter band witnesses a larger contribution of the π-back-donation from the Ti d orbitals to the π* orbital of CO, meaning that the polymer chains have a higher electron-releasing effect than the initial ethyl group. Moreover, both bands at 2072 and 1620 cm^–1^ are more intense in the present experiment, at equal optical thickness of the samples. This observation suggests that during ethylene polymerization more Ti^3+^ sites become accessible to incoming molecules, in good agreement with the results of kinetic experiments.^53^

**Figure 3:**
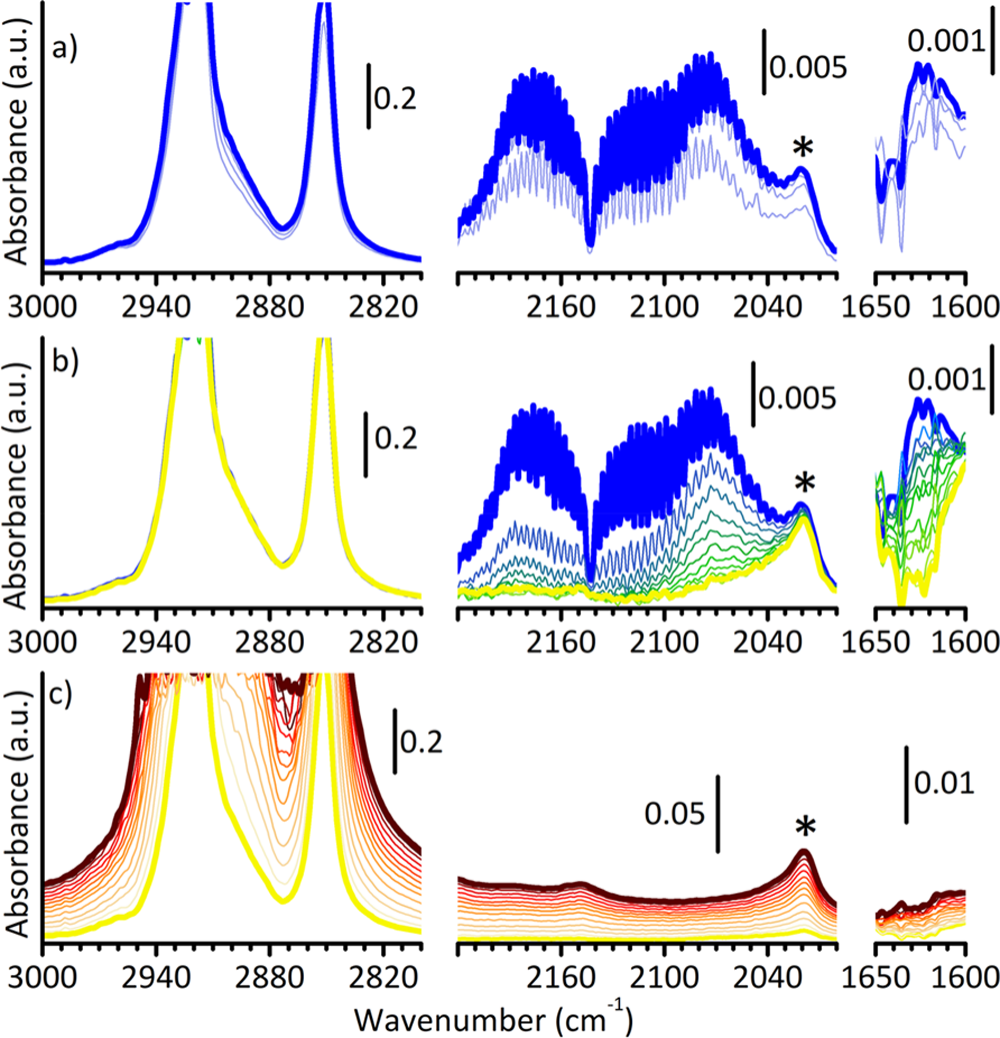
(a) FT-IR spectra collected as a function of time (up to bold spectrum in blue) during exposure of MgCl_2_/TiCl_4_/TEAl to a 1:1 mixture of C_2_H_4_/CO (equilibrium pressure = 100 mbar) at room temperature. The last spectrum is collected after 40 s. (b) FT-IR spectra collected during the subsequent controlled degassing of the reaction cell at room temperature (from θ_max_ in blue to θ_min_ in yellow). (c) FT-IR spectra collected as a function of time during exposure of MgCl_2_/TiCl_4_/TEAl to pure ethylene (equilibrium pressure = 50 mbar), from yellow to brown. The last spectrum is collected after 5 min. All the spectra are shown after subtraction of the spectrum of MgCl_2_/TiCl_4_/TEAl catalyst, in three different spectral regions: the ν(CH_2_) in the left (for PE), the ν(C≡O) in the middle (for adsorbed CO), and the ν(C=O) in the right (for Ti—acyl). The band indicated with an asterisk is due to PE (combination of wagging modes).^52^

Figure 3b shows the evolution of the main spectral features during the subsequent degassing in a controlled way. Both the bands at 2072 and 1620 cm^–1^ gradually decrease in intensity up to disappearance, while the spectroscopic manifestations of PE remain unaltered. Upon dosing back pure ethylene (*P*_C_2_H_4__ = 50 mbar), the polymerization reaction starts again and proceeds at constant speed for the successive 5 min, until the experiment was interrupted (Figure 3c). The experiment demonstrates that the poisoning effect of CO is reversible, in agreement with the industrial finding,^26^ thus confirming that the results obtained in this work at lab-scale and on a model Ziegler–Natta catalyst are actually representative for the real catalytic process.

All in all, the whole set of spectroscopic data discussed in this work finally fulfills the lack of direct experimental evidence for the interaction between CO and the Ti active sites in Ziegler–Natta catalysts, that was so far just hypothesized on the basis of indirect proofs coming from kinetic experiments and from the analysis of the CO-quenched polymers. Indeed, we have been able to detect the CO coordinated to alkylated Ti^3+^ sites and the Ti—acyl species formed upon subsequent insertion of CO into the Ti^3+^—alkyl bond. In more detail, we have found that both species are sensible to the intrinsic properties of the catalytic site and to the reaction environment and that both species are reversible upon decreasing the CO pressure, the former faster than the latter. Therefore, our study, carried out on a model Ziegler–Natta catalyst, demonstrates that FT-IR spectroscopy of CO adsorption is a powerful characterization technique for both monitoring the distribution of the active sites and inspecting their intrinsic properties, thus disclosing an effective and easily affordable way to investigate the more complex industrial Ziegler–Natta catalysts, under different working conditions.

## Supporting Information Available

Experimental details about the characterization technique and the working procedures (PDF)
