# Supplementary material for: Disclosing the Interaction between Carbon Monoxide and Alkylated Ti3+ Species: a Direct Insight into Ziegler–Natta Catalysis
Source: J Phys Chem Lett. 2020 Jun 25;11(14):5632–7. doi: 10.1021/acs.jpclett.0c01665 (PMC8008442; doi:10.1021/acs.jpclett.0c01665)
Supplement: Supplementary file 1 — jz0c01665_si_001.pdf [file jz0c01665_si_001.pdf]

Disclosing the Interaction between Carbon Monoxide and Alkylated Ti^{3+} Species: a Direct Insight into Ziegler-Natta Catalysis

Alessandro Piovano,* Jelena Zarupski, Elena Groppo

Department of Chemistry, INSTM and NIS Centre, University of Torino, Via Giuria 7, 10125 Torino, Italy

DPI, P.O. Box 902, 5600 AX Eindhoven, the Netherlands

* alessandro.piovano@unito.it

SUPPORTING INFORMATION

EXPERIMENTAL DETAILS

Characterization technique

FT-IR spectra were collected in transmission mode at a resolution of 2 cm^{-1} with a Bruker Vertex70 instrument (MCT detector). The samples were measured in the form of thin self-supporting pellets (surface density of ca. 40 mg cm^{-2}) placed inside a quartz cell equipped with two KBr windows, which allows performing thermal treatments and measurements in presence of gases. In order to monitor *in situ* the evolution of the spectra during the reactions, the quartz cell interfaced with the spectrophotometer was directly connected to a vacuum line for the regulation of the atmosphere on the sample.

Working procedures

Both ethylene polymerization and CO adsorption were performed directly inside the IR cell used for the catalyst synthesis. In the first case, 50 mbar of ethylene were dosed on the $MgCl_2/TiCl_4$/TEAl catalyst. The polymerization reaction started immediately, and it was monitored continuously by FT-IR spectroscopy with a time resolution of 10 seconds. As for CO adsorption, two types of experiments were performed. In the first one, 50 mbar of CO were dosed at room temperature and the sample was cooled down to 100 K by liquid nitrogen. A FT-IR spectrum was collected at 100 K, and then the CO pressure was progressively decreased through controlled expansions. A series of FT-IR spectra were collected at decreasing CO coverages and at constant temperature. In the second experiment, 50 mbar of CO were dosed on the sample at room temperature, followed by step-by-step degassing. A series of FT-IR spectra were collected at decreasing CO coverages.
